# Body odour disgust sensitivity predicts authoritarian attitudes

**DOI:** 10.1098/rsos.171091

**Published:** 2018-02-28

**Authors:** Marco Tullio Liuzza, Torun Lindholm, Caitlin B. Hawley, Marie Gustafsson Sendén, Ingrid Ekström, Mats J. Olsson, Jonas K. Olofsson

**Affiliations:** 1 Department of Psychology, Stockholm University, Stockholm, Sweden; 2 Department of Surgical and Medical Sciences, ‘Magna Graecia’ University of Catanzaro, Catanzaro, Italy; 3 Department of Psychology, ‘Sapienza’ University of Rome, Rome, Italy; 4 IRCCS Santa Lucia Foundation, Rome, Italy; 5 Department of Clinical Neuroscience, Karolinska Institutet, Stockholm, Sweden; 6 Swedish Collegium of Advanced Study, Uppsala, Sweden

**Keywords:** olfaction, behavioural immune system, authoritarianism, body odours, social conservatism

## Abstract

Authoritarianism has resurfaced as a research topic in political psychology, as it appears relevant to explain current political trends. Authoritarian attitudes have been consistently linked to feelings of disgust, an emotion that is thought to have evolved to protect the organism from contamination. We hypothesized that body odour disgust sensitivity (BODS) might be associated with authoritarianism, as chemo-signalling is a primitive system for regulating interpersonal contact and disease avoidance, which are key features also in authoritarianism. We used well-validated scales for measuring BODS, authoritarianism and related constructs. Across two studies, we found that BODS is positively related to authoritarianism. In a third study, we showed a positive association between BODS scores and support for Donald Trump, who, at the time of data collection, was a presidential candidate with an agenda described as resonating with authoritarian attitudes. Authoritarianism fully explained the positive association between BODS and support for Donald Trump. Our findings highlight body odour disgust as a new and promising domain in political psychology research. Authoritarianism and BODS might be part of the same disease avoidance framework, and our results contribute to the growing evidence that contemporary social attitudes might be rooted in basic sensory functions.

## 1. Introduction

Recent findings have suggested that human behaviour is governed by a ‘behavioural immune system’ (BIS) [1,2], a set of psychological mechanisms that presumably evolved in order to (i) detect cues of pathogen threats to health and survival, (ii) activate the appropriate affective and cognitive responses to such threats and (iii) trigger the relevant avoidance behaviours [3]. Disgust is a pervasive emotion that might have thus evolved as a defence mechanism to protect the body from contamination by potentially harmful substances [4] and thus disgust plays a pivotal role in the BIS [5].

Disgusting substances (e.g. faeces, mucus and wounds) often contain pathogens [3]. Disgust is, however, also elicited by noncontagious cues, such as physical, moral or sexual deviations, suggesting that an overly sensitive behavioural defence creates physical and social distance to others who are perceived as carrying even the slightest risk of contagion, leading to social stigmatization [6]. Moreover, recent reviews show a substantial overlap between the neurophysiological basis of core disgust and moral disgust, which supports the notion that social and moral disgust may have evolved from core disgust [7].

Disgust may thus underlie avoidance behaviours towards individuals and groups that are perceived as foreign, strange, morally deviant or norm violating [8,9]. For instance, Faulkner *et al.* [10] found that higher levels of pathogen salience and perceived vulnerability to disease (PVD) [11] predicted negative attitudes towards unfamiliar outgroups.

In fact, feelings of disgust have been consistently linked to the stigmatization of ethnic [9,12] and sexual minorities [13]. On a cross-national level, the risk of being exposed to parasites was associated with cultural differences in the endorsement of traditional values, conformism and in the emphasis on the psychological distinction between ingroup and outgroup [14,15]. From a BIS perspective, prejudice can be seen as a social discriminatory behaviour partly motivated by the fact that pathogens represent an invisible threat and individuals with high levels of disgust sensitivity might be more likely to avoid foreign people, and to promote policies that avoid contact with them, because they are perceived as potentially spreading unfamiliar pathogens, or different hygienic or food habits [16].

So-called right-wing authoritarianism is a relatively common and stable ideological attitude [17,18] favouring a rigid social order, designated roles for different genders and ethnic groups and a punitive attitude to perceived social, legal and moral transgressions [19]. Such authoritarianism is viewed as an ideological expression of being highly vigilant to perceived threats [20]. It has been shown that people on the right of the political spectrum tend to display a negativity bias [21,22], namely more marked psychological and physiological responses to features in the environment that are negative.

Of particular interest from the perspective of the BIS framework, individuals with authoritarian attitudes report higher disgust sensitivity, and higher physiological reactivity to disgust-evoking and aversive images [23]. This has been interpreted as suggesting that authoritarianism could be characterized as a disease avoidance mechanism, because authoritarianism would motivate less exposure to unfamiliar environments and individuals, and thus less exposure to pathogenic threats [24].

Indeed, Navarrete & Fessler [12] found that disgust sensitivity and PVD correlates with ethnocentrism. Since disgust helps to prevent contamination [25], cultures with a high level of exposure to pathogens may also be more socially conservative [15]. The investigation of the link between disgust and political ideology has captured the attention of political scientists [26], who are increasingly recognizing the role played by basic emotions in shaping political discourse. Hatemi & McDermott [27] suggested that one of the most plausible mediating factors in relating ideology to disgust might be the higher emphasis that conservatives place on moral ‘purity’, which is associated with disease avoidance concerns [28].

Smells and tastes are arguably the most potent disgust signals, and, accordingly, it has been theorized that moral disgust is intimately linked to chemoreception [29]. Environmental cues of infectious risk are often mediated by malodours, rather than by visual cues [30], and cross-cultural investigations show that odours, and body odours in particular, are among the most widespread triggers of disgust [31,32]. Moreover, following experimental induction of an inflammatory agent, body odours become more aversive to the perceiver [33]. Indeed, pathogenic processes can dramatically influence volatile organic compounds of the human body [34]. Olfaction is thus a potent cue system for disease risk. Bodily substances such as sweat, saliva and faeces are considered among the most potent triggers of disgust [31] and are indeed often fraught with pathogens [5].

When exposed to pathogen-related body odours (e.g. faeces), people report being more willing to pursue pathogen avoidant behaviours (e.g. using a condom during sex) [35]. Disgust-evoking odour stimulation was shown to amplify the severity of moral condemnation of norm violations [36]. Furthermore, a genome-wide analysis on the possible genetic determinants of ideology has suggested that differences in a DNA region that includes several genes related to olfaction vary systematically across liberals and conservatives [37].

We hypothesized that body odour disgust sensitivity (BODS) would be linked to social attitudes that favour traditionalism and prevent contact with outgroups, and more generally, with groups perceived as deviant. Specifically, we predicted that higher levels of BODS would be associated with a more authoritarian disposition. Furthermore, we predicted that individuals with higher levels of BODS would show a preference for the political candidate in the US presidential election 2016 that appeared to attract the sympathies of authoritarian participants. Thus, in studies 1 and 2, we tested whether scores on the recently developed BODS scale would have a positive relationship with authoritarianism, independently from other general disgust sensitivity scales that do not focus on body odour disgust. We recently showed that BODS scores are a good indicator of disgust responses to actual body odours, and thus BODS is particularly useful in survey-based studies [38]. While the BODS scale can be used as either a uni-dimensional construct (overall body odour disgust) or a two-factorial scale (body odour disgust for self versus others), these two factors are highly correlated, and in most cases they show similar patterns of convergent and discriminant validity when compared to other scales [39]. We thus chose to initially use the scale as a uni-dimensional construct.

In study 3, we took advantage of the imminent US presidential election 2016 to test the hypothesis that high BODS scores would be related to political sympathies. Specifically, BODS was hypothesized to predict positive attitudes towards Donald Trump, a then presidential candidate whose campaign message had been characterized as authoritarian [40]. In all studies, we used Amazon Mechanical Turk for data collection, a method that has some benefits over laboratory experiments, including that large amounts of data can be rapidly collected online from geographically distributed individuals. Even though samples recruited through Amazon Mechanical Turk may differ from the general population [41], Mechanical Turk samples have been shown to be more diverse and representative than common convenience samples (e.g. college students) typically used in psychological research [42,43]. Most importantly, for the purpose of the present study, data from Mechanical Turk studies have been shown to be both psychometrically valid [44] and a valid tool for research on political ideology [45]. Furthermore, we note that our studies were designed with the primary aim to assess the associations between body odour disgust and specific social attitudes, and not to characterize any specific group of voters.

## 2. Study 1

### 2.1. Method

#### 2.1.1. Participants

Based on effect sizes reported in previous research [46], we aimed at collecting data from at least 160 participants in order to gain a power of 0.80. In order to maximize the variability of our sample, we did not restrict our recruitment to any particular geographical region. Eligible participants were Mechanical Turk workers who had a prior approval rate of at least 85%. The study was launched on the 29 April 2013, and all data were collected in about four days. The final sample included 201 participants (83 females; age, *M* = 33.13, s.d. = 10.32 years). Participants were paid $0.50 each for their participation.

Three participants (1.5%) had no higher education, 12 (6%) had received a High school diploma or equivalent, 34 (16.9%) had attended some college, 9 (4.5%) had received an Associate degree, 106 (52.7%) a Bachelor degree, 33 (16.4%) a Master degree, 2 (1%) a Professional degree and 2 (1%) a Doctorate degree. In terms of race/ethnicity, 99 (49%) defined themselves as Asian (mainly due to a high degree of participation from Mechanical Turk workers based in India), 86 (43%) as white, 10 (5%) as black/African American and 5 (2.5%) as Latino/Hispanic.

#### 2.1.2. Measures

The BODS scale includes six body odour sources (breath, axillary sweat, feet, faeces, urine and gas), each of which appears in two different contexts: internal source (own smell) and external source (the smell of an unfamiliar person). The BODS scale displays excellent internal consistency and strong convergence with existing disgust sensitivity scales, and a recent study showed that BODS, compared to the most commonly used disgust scales, had a stronger relationship with PVD [39]. Furthermore, BODS scores correlated strongly with self-reported disgust reactivity to sweat bio-samples, suggesting BODS is a valid assessment of body odour disgust [38].

The BODS scale items can be found in the Material component on Open Science Framework (OSF) repository: https://osf.io/4w2nh/.

In each of the 12 scenarios, participants rate to what extent the scenario elicits disgust, using a scale that ranges from 1 (not disgusting at all) to 5 (extremely disgusting). A high level of internal consistency was obtained for the BODS scale (Cronbach's *α* = 0.93).

We included a set of well-validated scales for measuring individual differences in social attitudes. We used the Right-Wing Authoritarianism (RWA) scale, a well-established assessment measuring authoritarian dispositions [19]. We used a validated version with fifteen items that did not reference specific minority populations, and hence avoided conflating authoritarianism with specific prejudice [47]. Participants reported their level of agreement with each statement on a seven-point scale ranging from 1 (totally disagree) to 7 (totally agree). We included the three domains of disgust scale (TDDS) [48], a scale that measures three distinct domains of disgust sensitivity (moral, sexual and pathogen). Each factor is represented by seven items measured on a one- to seven-point scale, ranging from 0 (not at all disgusting) to 6 (extremely disgusting). For all of these measures, a high internal consistency was obtained (Cronbach's *α* > 0.80).

As olfactory disgust is assumed to be a form of pathogen disgust, we included only the TDDS pathogen subscale as a control (TDDS-pat, e.g. ‘Sitting next to someone who has red sores on their arm’), in order to provide the closest and most relevant comparison to body odour disgust. Instead, subscales measuring sexual disgust (e.g. ‘Watching a pornographic video’) and moral disgust (e.g. ‘A student cheating to get good grades') were not included because they correlate only weakly with pathogen disgust [48]. While those with an authoritarian disposition might often react with disgust to moral [49] and sexual norm violations [50], these issues were not of relevance here. Instead, we focused on pathogen threat, which is not a topic included in authoritarianism assessments. It should be noted that the RWA scale used in this study has at least eight items that are somewhat related to sexuality (e.g. ‘Gods laws about abortion, pornography and marriage must be strictly followed before it is too late, violations must be punished’), morality and sexuality (e.g. ‘Facts show that we have to be harder against crime and sexual immorality, in order to uphold law and order’) or morality in general (e.g. ‘There are many radical, immoral people trying to ruin things; the society ought to stop them’), but no items that are directly related to pathogen concerns. Trivial effects due to thematic overlap would thus not influence relationships between RWA and pathogen-related variables. But olfaction might play a role in pathogen avoidance, and thus pathogen disgust was considered the best suited and strict control measure when investigating the link between olfactory disgust and authoritarianism.

Finally, right-wing authoritarianism must not be conflated with political conservatism. For this reason, in addition to RWA, participants were also presented with three items to assess their degree of liberalism versus conservatism in the social, moral or fiscal domains. These items were adapted from Helzer & Pizarro [51] (‘When it comes to social/moral/fiscal issues, how would you describe your political attitudes?’). Participants reported their responses on three separate seven-point scales ranging from 1 (very conservative) to 7 (very liberal). The responses were subsequently reverse-coded to make their interpretation coherent with the other scales (i.e. high scores = high level of conservatism).

Other measures of self-reported attitudes towards gender roles, hygiene, personality, ideological affiliation and self-reported measures of olfactory functions were collected for the purpose of another study and, therefore, not included in the current analysis. However, all items and data from the original survey can be retrieved online on the OSF repository at the following link: https://osf.io/c8xjy/.

Bayesian inferences on zero-order correlations were performed using the free statistical package JASP [52] using both Pearson’ *r* and Kendall's *τ*, as the latter is better suited for variables that are not normally distributed. This procedure allows us to assess the relative evidence (Bayes factor; BF) in favour of the alternative (BF_10_) or the null (BF_01_) hypothesis. We chose this approach because it allows comparing the relative strength of evidence in favour of competing hypotheses (i.e. H0: *ρ* = 0 versus H1: *ρ* ≠ 0) [53]. Instead of the JASP default prior (width = 1) on the alternative hypothesis regarding the parameters *ρ* and *τ*, we used a prior of width = 0.5, because 68% of the coefficients sampled from that distribution would fall between −0.25 and 0.25, similarly to a normal distribution centred on zero and with s.d. of 0.25. The choice of this value was informed by the overall meta-analytical effect size (≈0.25) found for the relationship between BIS measures and social conservatism [24].

We interpreted and labelled the sizes of BFs according to the recommendations of Raftery [54] as referred to by Jarosz & Wiley [55].

### 2.2. Results

In the electronic supplementary material we provide descriptive results for all variables, along with their distributions.

**Figure 1.**
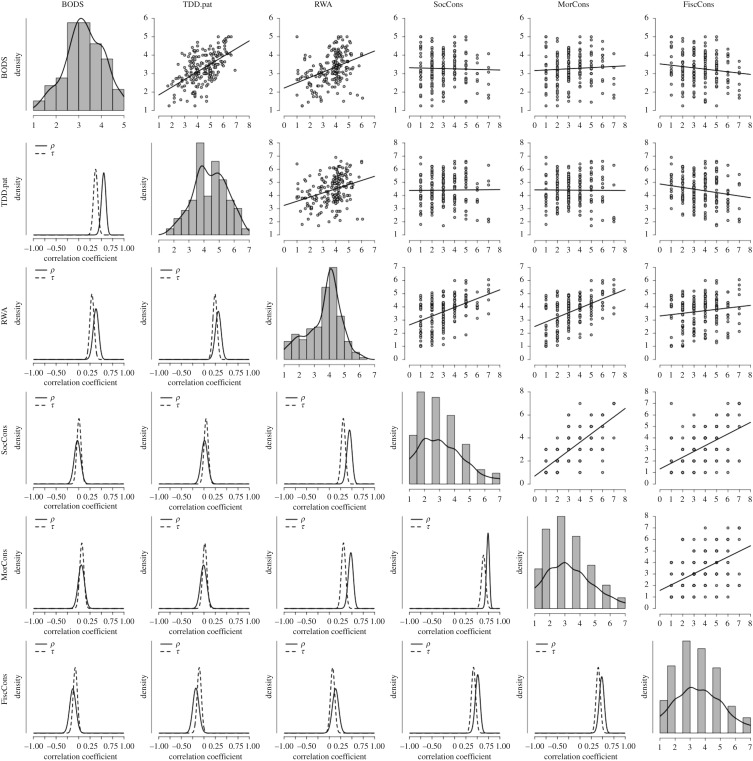
Correlation matrix for Study 1 data. On the top right are displayed the scatterplots and regression fit lines for the relationships among the variables measured in Study 1. Along the diagonal are displayed the histograms and density plots for each of the variables. On the bottom left are displayed the posterior distributions for the parameters *ρ* and *τ* under the alternative hypothesis using a *β* prior of width = 0.5. BODS, body odour disgust scale; TDD.pat, pathogen subscale of the three domains of disgust; RWA, right wing authoritarianism; SocCons, social conservatism; MorCons, moral conservatism; FiscCons, fiscal conservatism.

Consistent with our predictions, we found very strong evidence in favour of the hypothesis that RWA correlates with pathogen-related disgust (TDDS-pat, Pearson's *r* = 0.32, BF_10 _= 4620) and olfactory body odour disgust (Pearson's *r* = 0.39, BF_10 _= 995 802; see zero-order correlations in table 1 and figure 1).

**Table 1.** Correlation matrix of study 1. Pearson's *r* and Kendall's *τ* coefficients for the relationships among the variables measured in study 1 along with the Bayes factor in favour of the alternative hypothesis (*ρ* ≠ 0 and *τ*≠ 0) using a *β* prior of width = 0.5. BODS, body odour disgust scale, TDD.pat, pathogen subscale of the three domains of disgust; RWA, right wing authoritarianism; SocCons, social conservatism; MorCons, moral conservatism; FiscCons, fiscal conservatism.

		TDD.pat	RWA	SocCons	MorCons	FiscCons
BODS	Pearson's *r*	0.56	0.39	−0.03	0.06	−0.13
	BF_10_	9.859 × 10^14^	995 802	0.14	0.19	0.74
	Kendall's *τ*	0.38	0.30	0.01	0.07	−0.08
	BF_10_	1.118 × 10^13^	5.484 × 10^7^	0.14	0.42	0.51
TDD.pat	Pearson's *r*	—	0.32	0.01	−0.01	−0.18
	BF_10_	—	4620	0.13	0.13	3.48
	Kendall's *τ*	—	0.25	0.05	0.02	−0.11
	BF_10_	—	87 865	0.23	0.15	1.68
RWA	Pearson's *r*		—	0.45	0.48	0.14
	BF_10_		—	5.921 × 10^8^	1.880 × 10^10^	0.88
	Kendall's *τ*		—	0.32	0.32	0.07
	BF_10_		—	3.991 × 10^8^	7.289 × 10^8^	0.46
SocCons	Pearson's *r*			—	0.75	0.52
	BF_10_			—	1.525 × 10^33^	1.702 × 10^12^
	Kendall's *τ*			—	0.66	0.43
	BF_10_			—	2.072 × 10^40^	3.104 × 10^16^
MorCons	Pearson's *r*				—	0.49
	BF_10_				—	4.177 × 10^10^
	Kendall's *τ*				—	0.42
	BF_10_				—	2.672 × 10^15^

To establish the specificity of BODS in predicting RWA, we ran a series of Bayesian hierarchical multiple regressions where the parameters were estimated via quadratic approximation through the maximum *a posteriori* function implemented in the package *rethinking* [56].

Following the analytical strategy proposed by Richard McElreath [57] we used the widely applicable information criterion (WAIC) [58] to compare and average across models to account for uncertainty across models [56]. In fact, the WAIC can be conceptualized as an approximate Bayesian cross-validation [57].

We defined four models that included either the BODS, the TDDS-pat, both or neither of the two (table 2). We always included education, ethnicity, gender and age as possible confounding variables. All the variables were standardized to ease interpretability and parameter estimation. Priors for the BODS and TDDS-pat coefficients were normally distributed centred on 0 and with s.d. = 0.25, consistent with the overall meta-analytic effect size (≈0.25) found for the relationship between BIS measures and social conservatism [24]. This procedure makes it unlikely to have an estimate that deviates too much from the typical effect size found in the field. Priors for coefficients of the covariates of no theoretical interest (i.e. demographic variables) were normally distributed centred on 0 and with s.d. = 1, reflecting our higher level of uncertainty on these parameters. Finally, we computed and report means and the 95% percentile intervals (PIs) to summarize the posterior distribution of the estimates [57].

**Table 2.** Study 1. Model comparison and posterior parameter estimates for the regression on RWA. The most accurate model is in bold. *B* = standardized coefficients; SE *B *= standard error of the coefficients; WAIC, widely applicable information criterion. dWAIC, difference in WAIC from the best fitting model. DSE, standard error of the dWAIC. TDD.pat, pathogen subscale of the three domains of disgust; BODS, body odour disgust scale; edu, education.

	*B*	SE *B*	WAIC	dWAIC	weight	dSE
*Step1*			500.11	4.67	0.06	6.54
gender (*F* = 1)	−0.09	0.06				
age	0.03	0.06				
edu	−0.02	0.06				
ethnicity (white = 1)	−0.57	0.06				
TDD.pat	—	—				
BODS	—	—				
*Step2*			500.27	4.84	0.06	4.54
gender (F = 1)	−0.06	0.06				
age	0.01	0.06				
edu	−0.01	0.06				
ethnicity (white = 1)	−0.53	0.07				
TDD.pat	0.09	0.07				
BODS	—	—				
*Step3*			**495**.**43**	**0**.**00**	**0**.**67**	**0**.**00**
gender (F = 1)	−0.07	0.06				
age	0.02	0.06				
edu	0.02	0.06				
ethnicity (white = 1)	−0.49	0.07				
TDD.pat	—	—				
BODS	0.17	0.06				
*Step4*			497.76	2.33	0.21	0.53
gender (F = 1)	−0.07	0.06				
age	0.01	0.06				
Edu	0.02	0.06				
ethnicity (white = 1)	−0.48	0.07				
TDD.pat	0.02	0.07				
BODS	0.16	0.07				

The model comparison showed that the model that includes only the BODS, controlling for the confounding variables, was the most accurate (table 2).

Congruent with our hypotheses, BODS scores were related to RWA even when averaging across models, as shown by the average BODS coefficient and related PIs from the posterior distributions (*B* = 0.14, 95% PI = [LL = 0, UL = 0.30]). The results hence show a link between RWA and BODS that was not shared by another, non-olfactory pathogen disgust assessment.

## 3. Study 2

After establishing a specific association between BODS and authoritarianism, we sought to replicate and extend these findings in a sample of participants that was geographically restricted to the USA. Study 2 thus aimed to validate the hypothesized association of BODS and authoritarianism, and to compare the predictive utility of BODS to some other measures that have been previously associated with authoritarianism (see [24] for a review). In particular, we focused on overall disgust sensitivity [59,60] and germ aversion, assessed with the germ aversion subscale of the PVD scale [11].

### 3.1. Method

#### 3.1.1. Participants

Based on effect sizes reported in previous research [46], we aimed at collecting data from about 160 US participants in order to gain a power of 0.80. Eligible participants were Mechanical Turk workers who were based in the USA and who had a prior approval rate of at least 85%. The study was launched on 25 March 2015, and all data were collected in about six days. Participants were paid $0.50 each for their participation.

Six participants (3.6%) failed to respond correctly to the control question (‘To let us know that you're reading carefully, please check ‘Other’ below and write ‘I read it’ in the text box’) and were therefore removed from further analyses. The final sample consisted of 159 participants (75 females; age, *M*  =  35.11, s.d. = 11.88 years).

Three participants (1.9%) did not receive any higher education, 12 (7.5%) had received a High school diploma or equivalent, 45 (28.3%) had attended some college, 22 (13.8%) had received an Associate degree, 55 (34.6%) a Bachelor degree, 17 (10.7%) a Master degree, 4 (2.5%) a Professional degree and 1 (0.6%) a Doctorate degree. In terms of race/ethnicity, 133 (84%) defined themselves as white, 10 (6.3%) as Asian, 15 (9.5%) as black/African American and 8 (5%) as Latino/Hispanic.

#### 3.1.2. Measures

In this study we used some of the measures used in Study 1 (RWA, BODS and TDDS-pat) and added the disgust scale revised (DS-R) and the PVD scale. All of these measures displayed a Cronbach's *α* of at least 0.79. As in Study 1, our analysis strategy was to investigate the specificity and fit of a model in which BODS predicted authoritarianism.

The DS-R [60] is an updated version of the most well-established scale to measure individual differences in disgust sensitivity [59]. Participants indicated their agreement with 13 statements (e.g. ‘I never let any part of my body touch the toilet seat in a public washroom’) on a seven-point scale ranging from 1 (strongly disagree) to 7 (strongly agree), and rated how disgusting they would find 12 specific situations (e.g. ‘You see maggots on a piece of meat in an outdoor garbage pail’) on a seven-point scale ranging from 1 (not disgusting) to 7 (very disgusting). Although the scale includes a subscale assessing contamination-related disgust, it showed suboptimal internal consistency (Cronbach's *α* = 0.55), with a subsequent increase in the noise of the measurement and loss in statistical power [61]. Furthermore, while the TDDS subscales are theoretically motivated [48], the subscales of the DS-R are data-driven [60] and their boundaries are not always well-defined. For instance, while items such as item 4 (I never let any part of my body touch the toilet seat in a public washroom) are surely the expression of a strong concern of contamination, some items that are categorized as ‘core disgust’ (e.g. item 18, ‘You are about to drink a glass of milk when you smell that it is spoiled’) or ‘animal reminder’ (e.g. item 11, ‘It would bother me tremendously to touch a dead body’) are nonetheless likely motivated by pathogen avoidance concerns. We thus chose to use data from all items of the DS-R (Cronbach's *α* = 0.86), as it constitutes the most well-established general measure of disgust.

The PVD is a 15-item questionnaire, divided in two subscales, assessing individuals' discomfort in situations that imply a high likelihood of pathogen transmission (Germ Aversion subscale, e.g. ‘I don't like to write with a pencil someone else has obviously chewed on') and individuals' explicit beliefs that they are susceptible to contracting infectious diseases (Perceived Infectability subscale, e.g. ‘I am more likely than the people around me to catch an infectious disease’). Participants indicated their agreement on a seven-point scale (1 = strongly disagree; 7 = strongly agree, Cronbach's *α* = 0.88). We focused our analysis only on the Germ Aversion avoidance subscale (Cronbach's *α* = 0.79), as this is the subscale that relates more closely to the BIS [24].

Other measures of self-reported attitudes towards gender roles, hygiene, personality and ideological affiliation and self-reported measures of olfactory functions were collected for the purpose of another study and, therefore, not included in the current analysis. All the items and the data from the original survey can be retrieved online in the OSF repository at the following link: https://osf.io/c8xjy/.

Bayesian inferences on zero-order correlations were performed using the free statistical package JASP [52] using both Pearson’ *r* and Kendall's *τ*, as the latter is better suited for variables that are not normally distributed. This procedure allows us to assess the relative evidence (Bayes factor) in favour of the alternative (BF_10_) or the null (BF_01_) hypothesis. We chose this approach because it allows comparing the relative strength of evidence in favour of competing hypotheses (i.e. H0: *ρ* = 0 versus H1: *ρ* ≠ 0) [53]. Instead of the JASP default prior (width = 1) on the alternative hypothesis regarding the parameters *ρ* and *τ*, we used a prior of width = 0.5, because 68% of the coefficients sampled from that distribution would fall between −0.25 and 0.25, similarly to a normal distribution centred on zero and with standard deviation of 0.25. The choice of this value was informed by the overall meta-analytic effect size (≈0.25) found for the relationship between BIS measures and social conservatism [24].

We interpreted and labelled the sizes of BFs according to the recommendations of Raftery [54] as referred to by Jarosz & Wiley [55].

### 3.2. Results

In the electronic supplementary material we provide descriptive results for all variables, along with their distributions.

**Figure 2.**
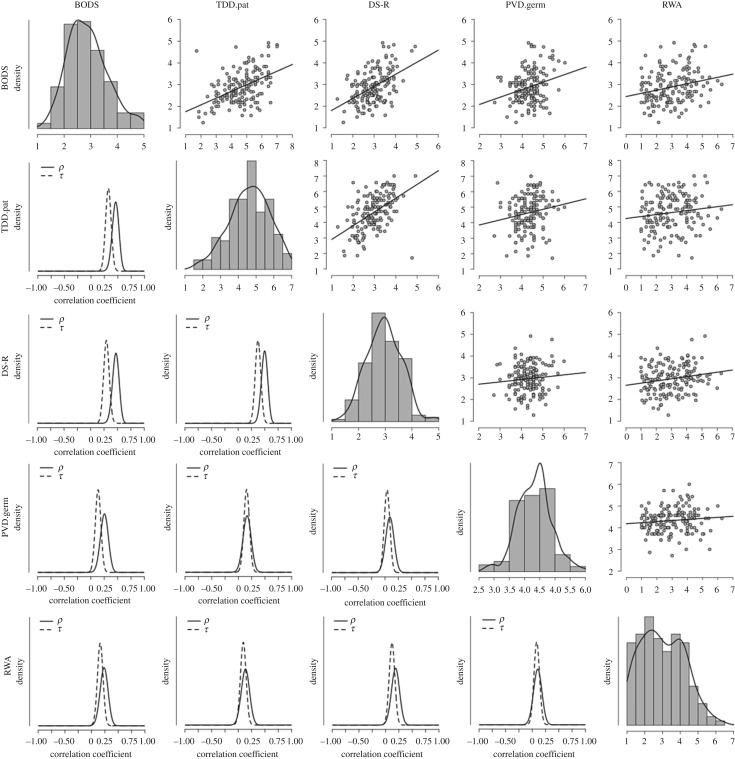
Correlation matrix for Study 2 data. On the top right are displayed the scatterplots and regression fit lines for the relationships among the variables measured in Study 3. Along the diagonal are displayed the histograms and density plots for each of the variables. On the bottom left are displayed the posterior distributions for the parameters *ρ* and *τ* under the alternative hypothesis using a *β* prior of width = 0.5. BODS, body odour disgust scale; TDD.pat, pathogen subscale of the three domains of disgust; DS-R, disgust sensitivity revised; PVD.germ, germ aversion subscale of the perceived vulnerability to disease; RWA, right wing authoritarianism.

Replicating findings from Study 1, we found positive evidence in favour of the hypothesis that RWA correlates with BODS (Pearson's *r *= 0.24, BF_10 _= 14.4) and weak evidence in favour of the correlation between RWA and general disgust sensitivity as measured by DS-R (Pearson's *r *= 0.19, BF_10 _= 2.5; table 3 and figure 2).

**Table 3.** Correlation matrix of Study 2. Pearson's *r* and Kendall's *τ* coefficients for the relationships among the variables measured on study 1 along with the Bayes factor in favour of the alternative hypothesis (*ρ* ≠ 0 and *τ* ≠ 0) using a *β* prior of width = 0.5. TDD.pat, pathogen subscale of the three domains of disgust; BODS, body odour disgust scale; DS-R, disgust sensitivity revised; PVD.germ, germ aversion subscale of the PVD; RWA, right wing authoritarianism.

		TDD.pat	DS-R	PVD.germ	RWA
BODS	Pearson's *r*	0.47	0.48	0.26	0.24
	BF_10_	2.795 × 10^7^	7.55 × 10^7^	30.08	14.44
	Kendall's *τ*	0.33	0.30	0.14	0.17
	BF_10_	3.000 × 10^7^	1.097 × 10^6^	4.08	19.43
TDD.pat	Pearson's *r*	—	0.51	0.17	0.14
	BF_10_	—	1.023 × 10^9^	1.32	.64
	Kendal's *τ*		0.39	0.16	0.10
	BF_10_	—	1.621 × 10^10^	12.58	0.75
DS-R	Pearson's *r*		—	0.09	0.19
	BF_10_		—	0.29	2.51
	Kendall's *τ*		—	0.04	0.12
	BF_10_		—	0.23	2.12
PVD.germ	Pearson's *r*			—	0.11
	BF_10_			—	0.35
	Kendall's *τ*			—	0.09
	BF_10_			—	0.59

In order to test the best predictor of authoritarianism among the two disgust measures that were most clearly related to RWA (BODS, DS-R), we entered these variables—along with age, gender, education and ethnicity—in a series of Bayesian hierarchical models compared and averaged through WAIC as described in Study 1. The model comparison showed that there is a certain amount of uncertainty (dSE is bigger than dWAIC) on what should be considered the best model in terms of WAIC among the ones that included the BODS, the DS-R or both (table 4). Although the posterior distributions PIs of both DS-R and BODS included zero in the averaged model, it should be noted that they correlate substantially and thus it might be that their joint contribution is crucial in explaining variance in RWA, but neither of the two seem to be a strong independent predictor of authoritarianism when controlling for the other.

**Table 4.** Study 2. Model comparison and posterior parameter estimates for the regression on RWA. The most accurate model is in bold. *B*, standardized coefficients; SE *B*, standard error of the coefficients, WAIC, widely applicable information criterion. dWAIC difference in WAIC from the best fitting model. dSE, standard error of the dWAIC. DS-R, disgust sensitivity revised; BODS, body odour disgust scale; edu, education.

	*B*	SE *B*	WAIC	dWAIC	weight	dSE
*Step1*			444.63	4.92	0.03	5.37
gender (*F* = 1)	0.17	0.08				
age	0.08	0.08				
edu	−0.21	0.08				
ethnicity (white = 1)	−0.13	0.08				
TDD.pat	—	—				
BODS	—	—				
*Step2*			**439**.**72**	**0**.**00**	**0**.**39**	—
gender (*F* = 1)	0.22	0.08				
age	0.08	0.08				
edu	−0.19	0.08				
ethnicity (white = 1)	−0.08	0.08				
DS-R	0.19	0.08				
BODS	—	—				
*Step3*			**440**.**90**	**1**.**18**	**0**.**21**	**5.07**
gender (*F* = 1)	0.17	0.08				
age	0.08	0.08				
edu	−0.18	0.08				
ethnicity (white = 1)	−0.08	0.08				
DS-R	—	—				
BODS	0.17	0.08				
*Step4*			**439**.**83**	**0**.**11**	**0**.**37**	**2.53**
gender (*F* = 1)	0.21	0.08				
age	0.08	0.08				
edu	−0.18	0.08				
ethnicity (white = 1)	−0.06	0.08				
DS-R	0.14	0.08				
BODS	0.11	0.08				

## 4. Study 3

In studies 1 and 2 we showed a consistent relationship between BODS and authoritarianism. A possible limitation from studies 1 and 2 is that these studies were conducted on the same samples used in a prior validation study [39], so we felt compelled to replicate the results in a new sample. Furthermore, the US presidential elections 2016 offered a unique opportunity to test the ability of the BODS to predict attitudes towards the political candidates, in particular Donald Trump, who was described as having a particularly authoritarian message [40].

We hypothesized there would be a positive correlation between BODS scores and positive attitudes towards Trump, but we also expected this relationship to be explained by RWA, a variable that we hypothesized would be positively associated with both BODS scores and support for Trump. We expected this mediating role of RWA to not be shared with measures that reflect an endorsement of social dominance or inequality [20], such as social dominance orientation (SDO) [62,63]. While RWA and SDO are positively correlated in the population, SDO uniquely captures the tendency of some individuals to ‘step on others to get ahead’, is motivated by perceiving the world as a ‘competitive jungle’ and prompts dominance behaviours towards groups perceived as competing for resources, etc. On the other hand, RWA is motivated by perceiving the world as a dangerous place and prompts prejudice towards threatening groups, and RWA is therefore more likely to be related to threat-avoidance concerns, such as those involving pathogens.

### 4.1. Method

#### 4.1.1. Participants

We established a sample size that would allow us to detect, with 80% power, a relationship between RWA and BODS that was half as strong as that found on average in Studies 1 and 2 (*r* = 0.16). A power analysis yielded at least 313 participants. To prevent the possible impact of high drop-out rates, our survey was set to aim for 400 participants. Eligible participants were Mechanical Turk workers who were based in the USA and who had a prior approval rate of at least 85%.

Study 3 was launched on 12 October 2016 (about one month prior to the presidential election), and all data were collected in about 24 h. A final sample of 391 participants (219 females; age, *M* = 37.45, s.d. = 12.19 years) was recruited from the USA using through Amazon Mechanical Turk. Participants were paid $0.50 each for their participation.

Three participants (0.8%) did not receive any higher education, 45 (11.5%) had received a High school diploma or equivalent, 95 (24.3%) had attended some college, 46 (11.8%) had received an Associate degree, 141 (36.1%) a Bachelor degree, 49 (12.5%) a Master degree, 5 (1.3%) a Professional degree and 7 (1.8%) a Doctorate degree. In terms of race/ethnicity, 305 (78%) defined themselves as white, 23 (5.9%) as Asian, 22 (5.6%) as black/African American, 4 (1%) as native American and 1 (less than 1%) as Latino/Hispanic.

#### 4.1.2. Measures

We included the RWA and BODS assessments similarly to Studies 1 and 2, and added SDO as a control measure. All of these measures displayed a Cronbach's *α* of at least 0.93. The SDO measures the tolerance for inequality among social groups and in this study we used a well-established 16-item version [62]. Participants were asked to indicate how they felt about each of the 16 statements (e.g. ‘It's OK if some groups have more of a chance in life than others.’) on a 1 (very negative) to 7 (very positive) Likert-type scale.

Additionally, in order to assess participants' attitudes towards each of the five then presidential candidates (Donald Trump, Hillary Clinton, Gary Johnson, Jill Stein and Darrel Castle), we adapted items from the American National Election Studies [64] that assessed, through a visual analogue scale from 0 to 100:

— two items assessing the Emotional Thermometer towards each: (i) ‘The US presidential elections are approaching. How would you feel about each candidate becoming the next president of the USA?’ with a response setting ranging from 0 (extremely negative) to 100 (extremely positive), and (ii) ‘We'll show the name of a person or group and we'd like you to rate that person or group using something we call the feeling thermometer’ with a response setting ranging from 0 (very cold or unfavourable feeling) to 100 (very warm or favourable feeling).
— One item assessing participants' voting intentions (i.e. ‘How likely are you to vote for each presidential candidate?’) with a response setting ranging from 0 (extremely unlikely) to 100 (extremely likely).

We narrowed our analysis down to the two candidates who were leading the polls at the time when the study was conducted, namely Hillary Clinton (Democrat) and Donald Trump (Republican). We found that the attitudes and voting intentions measures displayed high internal consistency (Cronbach's *α* ≥ 0.96), and collapsed them to an overall ‘support’ variable for each candidate.

Other measures of self-reported attitudes towards gender roles and ideological affiliation were collected for the purpose of another study and therefore not included in the current analysis.

Method and statistical analysis package were identical to those used in Study 2. Our prior of width = 0.5 was based on the overall meta-analytical effect size (≈0.25) found for the relationship between BIS measures and social conservatism [24].

We interpreted and labelled the sizes of BFs according to the recommendations of Raftery [54] as referred to by Jarosz & Wiley [55].

### 4.2. Results

In the electronic supplementary material we provide descriptive results for all variables, along with their distributions.

**Figure 3.**
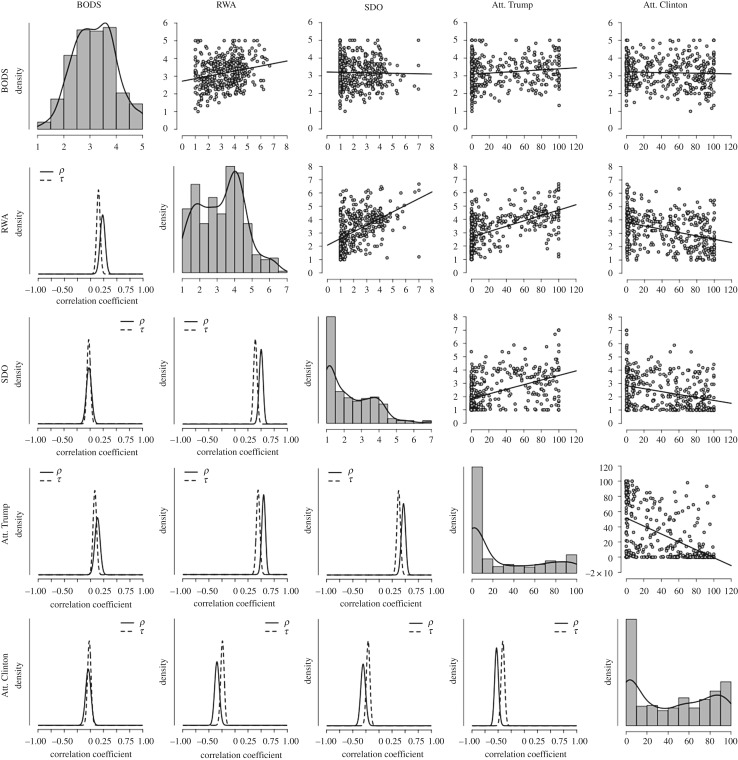
Correlation matrix for Study 3 data. On the top right are displayed the scatterplots and regression fit lines for the relationships among the variables measured in Study 2. Along the diagonal are displayed the histograms and density plots for each of the variables. On the bottom left are displayed the posterior distributions for the parameters *ρ* and *τ* under the alternative hypothesis using a *β* prior of width = 0.5. BODS, body odour disgust scale; RWA, right wing authoritarianism; SDO, social dominance orientation; Att. Clinton, positive attitudes towards Hillary Clinton; Att. Trump, positive attitudes towards Donald Trump.

Both RWA and SDO were related to positive attitudes towards Trump (table 5 and figure 3, BFs_10 _> 1 000 000). Replicating findings from Studies 1 and 2, we found very strong evidence in favour of the hypothesis that BODS correlates with RWA (Pearson's *r *= 0.23, BF_10 _= 3977). However, we also found positive evidence in favour of the absence of a relationship between BODS and SDO (*r *= −0.02, BF_01_ = 9.5). We found weak evidence in favour of the hypothesis that BODS correlates with positive attitudes towards Trump (*r* = 0.13, BF_10_ = 2.8). Although the evidence in favour of this relationship is weak, it should be noted that when controlling for the other demographic variables through a Bayesian regression model and a narrower prior distribution of the parameters (the same as those used in Studies 1–2), the relationship between the BODS scores and attitudes towards Trump was found to be confidently above zero (*B *= 0.17, 95% PI = [0.08, 0.27]). Overall, this result suggests that, when controlling for other possible confounding variables and having a prior belief that is informed by previous literature, we can confidently believe that attitudes toward Trump were positively related to BODS scores, given the data, although the effect size was small.

**Table 5.** Correlation matrix of Study 3. Pearson's *r* and Kendall's *τ* coefficients for the relationships among the variables measured on study 1 along with the Bayes factor in favour of the alternative hypothesis (*ρ* ≠ 0 and *τ* ≠ 0) using a *β* prior of width = 0.5. BODS, body odour disgust scale; RWA, right wing authoritarianism; SDO, social dominance orientation; Att. Clinton, positive attitudes towards Hillary Clinton; Att. Trump, positive attitudes towards Donald Trump.

		RWA	SDO	Att. Trump	Att. Clinton
BODS	Pearson's *r*	0.23	−0.02	0.13	−0.04
	BF_10_	3977	0.11	2.82	0.13
	Kendall's *τ*	0.15	−0.03	0.08	−0.02
	BF_10_	1947	0.14	1.80	0.12
RWA	Pearson's *r*	—	0.51	0.55	−0.35
	BF_10_	—	3.406 × 10^23^	1.725 × 10^29^	5.479 × 10^9^
	Kendall's *τ*	—	0.40	0.45	−0.24
	BF_10_	—	1.814 × 10^28^	3.812 × 10^36^	1.097 × 10^10^
SDO	Pearson's *r*		—	0.47	−0.31
	BF_10_		—	5.245 × 10^19^	1.275 × 10^7^
	Kendall's *τ*		—	0.38	−0.21
	BF_10_		—	5.797 × 10^25^	1.529 × 10^7^
Att. Trump	Pearson's *r*			—	−0.54
	BF_10_			—	1.179 × 10^27^
	Kendall's *τ*			—	−0.416
	BF_10_			—	2.181 × 10^31^

We tested whether the coefficient of the relationship between SDO and BODS is smaller than the coefficient of the relationship between RWA and BODS even when taking other possible confounding variables into account. To this purpose, we fitted our data in a Bayesian regression model as in the previous analyses and computed the mean and PIs of the difference between coefficients from the posterior distributions. We found that BODS is reliably more related to RWA than SDO (mean *B* difference = 0.40, 95% PIs = [0.22, 0.59]).

**Figure 4.**
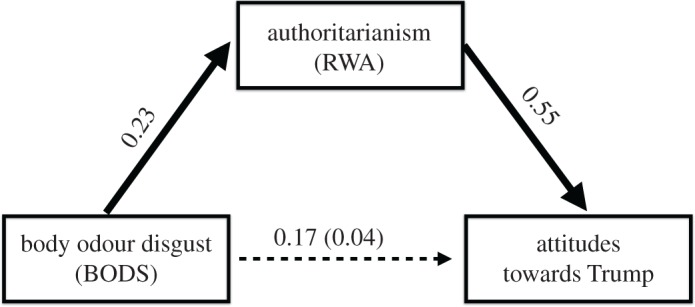
Study 3. Mediation analysis. Right wing authoritarianism (RWA) as a mediator of the effect of BODS on attitudes towards Donald Trump. Values represent standardized parameter estimates for each path. Along the path from BODS to attitudes towards Trump the numbers in parentheses represent the coefficients when RWA was entered into the analyses. The dashed line indicates that the direct path is significantly mediated by the indirect path (i.e. its estimated confidence intervals do not include zero).

Finally, we conducted mediational analyses on the data from Study 3, through the mediation R package [65] using a quasi-Bayesian Monte-Carlo method based on normal approximation [66], to test if the relationship between BODS and positive attitudes towards Trump was mediated by authoritarianism. As predicted, the results indicated that the relationship between BODS and attitudes towards Donald Trump was fully mediated by RWA (figure 4; 95% CI = [0.075, 0.19)].

## 5. Discussion

Although the BIS framework assumes a relationship between pathogen avoidance motives and an orientation towards authoritarianism and social dominance [24], the role played by body odours has been largely overlooked [39]. Odours originating from the human body are among the most potent triggers of disgust and having a strong body odour is considered socially stigmatizing [67]. Although traditional notions of human ‘pheromones’ have been criticized [68], body odours contain information about emotions [69,70] and health [33] (see [71] for a recent review). Indeed body odours are affected by pathological processes [34], and we found that individual differences in disgust sensitivity to body odours are related to perceived vulnerability to diseases to a greater extent than individual differences in general disgust sensitivity [39].

Across three studies, we provided evidence that high levels of disgust sensitivity for body odours predicts authoritarian attitudes that imply resistance to social change and motivates separation of groups and individuals. Importantly, BODS does not positively relate to self-reported measures of conservatism when dissected into moral, social and fiscal aspects (Study 1), so the link to body odour disgust appears specific to authoritarianism, although the use of single item measures of ideology poses obvious reliability concerns.

Although the size of the BODS--authoritarianism association is of small-to-medium size, its magnitude is in line with previous meta-analytical evidence (0.21 < *r*s < 0.36) on the relationship between BIS measures and social conservatism [24]. More generally, the effect sizes found in our three studies are consistent with the finding of a ‘meta-meta-analysis’ summarizing 322 meta-analyses with more than 25 000 published studies in the field of personality and social psychology [72], which showed an average effect of *r* = 0.21. Furthermore, although the idea that implicit motivations determine political attitudes is intriguing [73], they are arguably predominantly driven by explicit processes, where socialization plays an important role (see [74] for a detailed review). Hence it is not surprising to see implicit motivations explaining only a small portion of the overall variance when it comes to political attitudes.

Our observation that BODS relates more strongly to RWA than to SDO (Study 3) fits very well with the ‘dual process’ framework proposed by Duckitt [20], and that assumes different underlying motivations for authoritarianism and SDO. According to this view, authoritarian motives arise from the perception of a social threat, described by the metaphor of the world as a dangerous place, and leads to the upholding of social order and tradition. By contrast, social dominance is prompted by the perception of the world as a competitive jungle, where it is justified to step on others to get ahead. Thus, authoritarianism might be more closely linked to avoidance, whereas social dominance would be linked to competitiveness. Indeed, BODS and authoritarianism (but not social dominance) may covary because they can both be conceptualized as a defence against possible pathogen threats [75,76], coherent with the BIS framework [16]. In support of this interpretation, recent work found that cultural differences in the level of exposure to pathogens are related to authoritarianism, but not to social dominance [77]. Moreover, it has been found that intergroup contact might decrease intergroup hostility prompted by authoritarian motives, but not intergroup hostility prompted by dominance motivations [78]. Similarly, we may speculate that familiarization with odours associated with foreign cultures may reduce prejudice in participants high in authoritarianism, but not in social dominance.

Only a few studies have investigated the relationship between disgust sensitivity and voting preferences [79]. In Study 3, we found positive evidence in favour of a weak relationship between disgust sensitivity to body odours and attitudes toward the Republican candidate Donald Trump. Importantly, this relationship is fully explained by authoritarian attitudes which were stronger among participants supporting Trump, a result that confirms the notion that in our study sample, Donald Trump was capable of attracting the sympathies of authoritarian voters [40]. In fact, it can be argued that Trump's firm stance against immigration, especially from groups viewed as culturally unfamiliar, might meet an implicit need of protection from pathogen threats from people perceived as either potential carriers of unfamiliar pathogens, or groups whose behaviours in disease-avoidance relevant behaviours (e.g. hygiene or food preparation) was perceived as deviant [16]. Our findings suggest that high reactivity to pathogen threats signalled by body odours is part of an ideological disposition towards authoritarian candidates, because of the link between disease avoidance and authoritarianism.

The reason why the BIS is often hyper-vigilant is that bacterial threats are often not detectable through visible cues [80], so the system acts in accordance with a ‘better safe than sorry’ logic [81] to limit contact with unfamiliar environments and groups [82]. In fact, meta-analytical evidence showed a consistent association between different manifestations of social conservatism and individual differences in the activation of the BIS [24], although none of the studies included in this meta-analysis had a special focus on body odours disgust. Considering the central role played by olfaction in preventing microbial hazards [32] and the observation that body odours can signal the presence of a disease [33], it is perhaps not surprising that individual differences in body odour disgust sensitivity, reliably assessed with a well-validated questionnaire [38,39], is indeed a predictor of authoritarianism, an attitude suggesting avoidance of unfamiliar groups.

There are limitations to this study. Most importantly, our samples are not randomly drawn from the population, but are based on voluntary engagement of individuals within the online community of Mechanical Turk workers. It is well-known that in most psychological studies, whites and liberals are over-sampled relative to conservatives and minority populations [41]. However, our results show 36% of participants supporting Trump over Clinton, at a time when Trump was polling at around 40% of the popular vote [83], indicating our sample was representative in this regard. Furthermore, Mechanical Turk samples are known to be more diverse than laboratory studies conducted at universities. Future work should attempt to overcome the challenges associated with population-based sampling. A second limitation is that we did not measure disgust responses to actual odours, but used the body odour disgust scale (BODS) to probe differences in disgust sensitivity. However, we recently showed that BODS scores predict disgust responses to sweat samples [38] so our conclusions would likely generalize to situations involving odour stimulation.

Disgust sensitivity and authoritarian attitudes may vary as a function of parasite stress [15,77]. In our sample we were unable to address the levels of pathogen stress of the environments where our respondents had been living. Future cross-national investigations would benefit from integrating predictors of authoritarianism in local environments.

Another possible limitation is that we lacked a measure of income, which is often associated with positive attitudes toward immigration [84]. Nevertheless, in our analyses, we controlled for education, which may be considered a good proxy of income and other lifestyle factors.

There is increasing interest in the role of olfaction and disgust in social attitudes [26,27,37,85]. Our work complements these on-going efforts by providing a relevant measure (BODS scale) of the individual susceptibility for odour-evoked disgust and its links to social attitudes. This line of research might also further our understanding of how basic chemosensory processes might have evolved as triggers of disgust and social regulation mechanisms [75].

## Supplementary Material

Descriptive Statistics of the three studies

## Data accessibility

Raw data are stored at Dryad: (https://doi.org/10.5061/dryad.6t536) [86].

Materials, data, R scripts and JASP files for reproducing all the reported analyses and procedures are stored in the OSF repository, and publicly accessible at the address: https://osf.io/c8xjy/. A pre-print version of the manuscript was published on PsyArXiv: https://psyarxiv.com/xs3f4/ on 20 October 2017, with the following doi:10.17605/OSF.IO/XS3F4.
