# Supplementary material for: Body odour disgust sensitivity predicts authoritarian attitudes
Source: R Soc Open Sci. 2018 Feb 28;5(2):171091. doi: 10.1098/rsos.171091 (PMC5830723; doi:10.1098/rsos.171091)
Supplement: Descriptive Statistics of the three studies [file rsos171091supp1.pdf]

Body odor disgust sensitivity predicts authoritarian attitudes.

Supplementary Information.

	N	Mean	SD	Min	Median	Max	Skewness	Kurtosis
Age	199	33.13	10.37	19	30	68	7.13	3.12
Education	201	4.59	1.28	1	5	8	-3.87	0.66
TDD.pat	201	4.40	1.10	1.7	4.4	6.9	-0.55	-1.63
BODS	200	3.27	0.82	1.25	3.25	5	-0.73	-1.08
RWA	201	3.67	1.11	1	4	6.07	-3.49	-0.47
SocCons	201	3.13	1.51	1	3	7	3.14	-0.77
MorCons	201	3.31	1.52	1	3	7	2.52	-1.24
FiscCons	201	3.59	1.53	1	4	7	1.36	-1.64

Table S1. Descriptives of the measures used in Study 1.TDD.pat = Pathogen Subscale of the Three Domains of Disgust; BODS = Body Odor Disgust Scale; RWA = Right Wing Authoritarianism; SocCons = Social Conservatism; MorCons = Moral Conservatism; FiscCons = Fiscal Conservatism.

	N	Mean	SD	Min	Median	Max	Skewness	Kurtosis
Age	158	35.11	11.88	18	31	74	5.40	0.98
Education	159	4.17	1.36	1	4	8	-0.10	-1.29
DSR	159	2.95	0.64	1.28	2.96	4.92	0.63	-0.18
TDD.pat	159	4.64	1.13	1.71	4.71	7	-1.32	-0.95
PVD.germ	161	4.33	0.65	1	4.29	7	-1.86	12.49
BODS	159	2.88	0.75	1.25	2.82	4.92	2.63	-0.09
RWA	159	3.00	1.22	1	2.87	6.27	1.36	-1.92

Table S2. Descriptives of the measures used in Study 2. TDD.pat = Pathogen Subscale of the Three Domains of Disgust; BODS = Body Odor Disgust Scale; RWA = Right Wing Authoritarianism; DSR = Disgust Sensitivity Revised; PVD.germ = Germ aversion subscale of the Perceived Vulnerability to Disease.

	N	Mean	SD	Min	Median	Max	Skewness	Kurtosis
Age	391	37.45	12.19	19	35	75	6.97	0.31
EDU	391	6.23	1.41	3	7	10	0.38	-1.95
BODS	391	3.17	0.80	1	3.17	5	0.34	-1.24
RWA	391	3.26	1.30	1	3.4	6.67	0.93	-2.77
SDO	391	2.35	1.32	1	2	7	7.09	0.99
Attitudes.Trump	390	28.78	35.58	0	6.835	100	6.89	-3.55
Attitudes.Clinton	390	43.44	36.46	0	42.165	100	1.21	-6.21

Table S3. Descriptives of the measures used in Study 3. BODS = Body Odor Disgust Scale; RWA = Right Wing Authoritarianism; SDO = Social Dominance Orientation; Att. Clinton = Positive attitudes towards Hillary Clinton; Att. Trump = Positive attitudes towards Donald Trump.
